# Supplementary material for: Chromothripsis during telomere crisis is independent of NHEJ, and consistent with a replicative origin
Source: Genome Res. 2019 May;29(5):737–49. doi: 10.1101/gr.240705.118 (PMC6499312; doi:10.1101/gr.240705.118)
Supplement: Supplemental Material [file supp_gr.240705.118_Supplemental_file_1.zip › contigs/annotated_contigs/DB57/contig.2.DB57_length_839_mean_cov_9.11084624553.docx]

**DB57_length_839_mean_cov_9.11084624553**

```
GGTCCAGAAACAT|ATGAAAAAAAGCTCAACATCACTGAGCATTAGAGAAATGCAAATCAGAACCACAATGAGATACCATATCATGCCA
              >chrX:79316396-79316818 - E=8e-182 p=0e+00
GTCAGAAGGGCAATTATTAAAAAGTCAAGAAACAACAGATGCTGGTGAGGTGGTGGAGAAATAGGAACACTTTTACACTGTTGGTGGGA

GTGTAAACTAGTTCAACCATTGTGGAAGGCAGTGTGGTGATTCTTCAAGGATCTAGAACTAGAAACACCATTTGACCCAGCCATCCCAT

TACTAGGTATATACCCAAAGGATTATAAATCATGCTAGTATAAAGACACATGCAGATGTATGTTTATTGTGGCAGTATTCACAATAGCA

AAGACTTCAAACCAGCCCAAATGTCCATCAATGATAGACTGGATTAAGAAAATATGGCACATATACATCAGGGAATACTA|CACT|AAA
                                                                                     >c
AAAAAAGATTATCAGCAGAGTAAACAGATGGCCTAGAGAATGAAATAAAATATTTGCAAAGGTACGTCCAACAAAGGTCCAATATCCAG
hrX:79315210-79315610 - E=1e-225
AGTCTATAAGAAATTGTTTTAAAATATACAAGCAAAAAACAAATAAACCCATTAAGAAGTGGACAAAGAACATGAGCAGACACTTCTGA

AAAGAAGACATGCAAGCATCTCACAAACATGAAAAATGCTCAGTCTCACTAACCATTTGAGAAATGCAAATCTAAACCACAATGAGATA

ACATCTCTCACCAGTCAGAATGGCTATTATTAAAAAGTAAACAAAAACAAAACAAACAATTAAACAAAACGTGGTACTGTTGTTGAATC

AAGTTTAGCCTAAAGCTGCCTCCTCACATACTTTATATTTG
```
